# Two New Chiratane-Type Triterpenoids from *Swertia kouitchensis*

**DOI:** 10.3390/molecules18078518

**Published:** 2013-07-18

**Authors:** Luo-Sheng Wan, Ting-Ting Liu, Xiao-Jun Lin, Qiu-Xia Min, Jia-Chun Chen

**Affiliations:** 1 Hubei Key Laboratory of Natural Medicinal Chemistry and Resource Evaluation, School of Pharmacy, Tongji Medical College, Huazhong University of Science and Technology, Wuhan 430030, China; 2 The First Affiliated Hospital, College of Medicine, Zhejiang University, Hangzhou 310003, China; 3 Shantou Institute for Drug Control, Shantou 515041, China; 4 State Clinical Research Center of TCM, Hubei 430061, China

**Keywords:** *Swertia kouitchensis*, diabetes, chiratane, triterpenoid, α-glucosidase

## Abstract

Two rare new chiratane-type triterpenoids, kouitchenoids A and B (**1**, **2**), together with oleanolic acid (**3**) and ursolic acid (**4**), were isolated from ethanol extract of *Swertia kouitchensis*. The new structures were determined by the analysis of MS and NMR data. In addition, compounds **1**–**4** showed moderate inhibitory activity against the α-glucosidase (with IC_50_ values ranging from 1,812 to 2,027 μM).

## 1. Introduction

**Figure 1:**
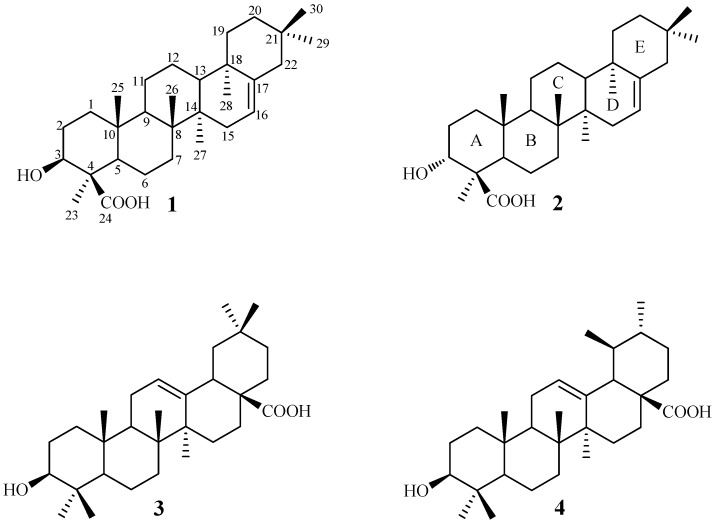
Structures of compounds **1**–**4**.

*Swertia kouitchensis* Franch. (Gentianaceae), widely distributed in China, has been used for the treatment of hepatitis and diabetes [1,2]. Previous study revealed that the ethanol extract of *S. kouitchensis* showed *α*-glucosidase inhibitory effect [3]. Thus, we initiated a study on the subject. As a result, two rare chiratane-type triterpenoids, kouitchenoids A and B (**1** and **2**), along with oleanolic acid (**3**) [4] and ursolic acid (**4**) (Figure 1) [5] were isolated and identified. All of these compounds were evaluated for their inhibitory activities against *α*-glucosidase. Described herein are the isolation, structure elucidation, and biological activities of these compounds.

## 2. Results and Discussion

The 95% ethanol extract of *S. kouitchensis* whole plants was suspended in water and successively partitioned with petroleum ether, CH_2_Cl_2_, EtOAc and *n*-butanol. The CH_2_Cl_2_ fraction was subjected to column chromatography and partitioned as described in the Experimental section to afford two new triterpenoids **1** and **2**, along with two known compounds **3** and **4**.

**Figure 2:**
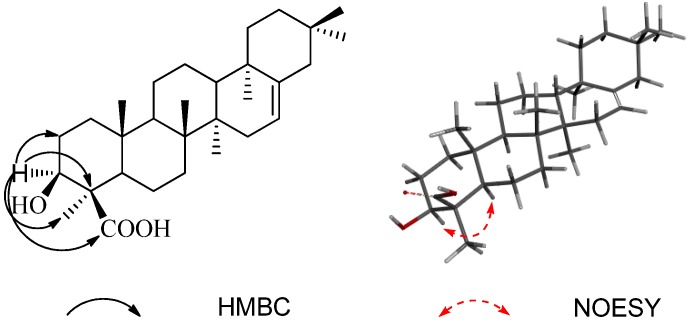
Key HMBC, and NOESY correlations of compound 1.

Compound **1** was obtained as white amorphous powder, gave a molecular formula of C_30_H_48_O_3_ by HRESIMS (*m/z* 455.3525 [M−H]^−^, calcd. for C_30_H_47_O_3_, 455.3531). Its IR spectrum exhibited absorptions at 3,453 and 1,696 cm^−1^, assignable to hydroxyl and carboxyl groups, respectively. The ^1^H- and ^13^C-NMR spectra of **1** (Table 1) closely resembled those of chiratenol [6,7], except for the appearance of a carboxyl carbon signal at *δ*_C_ 181.0 instead of a methyl (C-24) signal of chiratenol, and the downfield shift (+10.8 ppm) for C-4. The position of this carboxyl group was confirmed by HMBC spectrum (Figure 2), in which cross-peaks were observed between H-3 (*δ*_H_ 3.41) and C-2, C-4, C-23, and C-24, so that the carboxyl group is assigned to C-24. And also, H-3 was assigned as *α*-orientation by the NOESY correlation (Figure 2) between H-3 and H-5. Therefore, **1** was deduced to be 3*β*-hydroxy-chirat-16-en-24-oic acid, and named kouitchenoid A.

Compound **2** was obtained as white amorphous powder. Its molecular formula was assigned to be C_30_H_48_O_3_ based on the HRESIMS spectrum at *m/z* 455.3525 [M−H]^−^ (calcd. for C_30_H_47_O_3_, 455.3531). IR spectrum of **2** exhibited absorptions at 3,448 and 1,701 cm^−1^, assignable to hydroxyl and carboxyl functions, respectively. Its ^1^H-NMR and ^13^C-NMR shifts for the B, C, D, and E rings were almost the same as those of compound **1**, while those of the A ring differed. The main differences in the A ring between these two compounds were that the oxygenated proton H-3 at *δ*_H_ 3.41 (1H, *dd*, *J* = 12.0, 4.4 Hz) in **1** was changed into *δ*_H_ 4.75 (1H, *brs*, W_1/2_ = 5.7Hz) in **2** and the oxygenated C-3 carbon at *δ*_C_ 78.7 in **1** was shifted upfield to *δ*_C_ 71.1 in **2**, suggesting that the H-3 in a *β*-orientation in **2**. This assignment could be further supported by the missing correlation between H-3 and H-5 in the NOESY spectrum of **2**. Together with its ^1^H-^1^H COSY and HMBC spectra, which also were similar to those of **1**, compound **2** was determined to be 3*α*-hydroxy-chirat-16-en-24-oic acid and named kouitchenoid B.

**Table 1:** ^1^H-NMR (400 MHz) and ^13^C-NMR (100 MHz) Spectral Data of Compounds **1** and **2** in C_5_D_5_N (*δ* in ppm, *J* in Hz).

Position	1				2		
	*δ*_C_		*δ*_H_		*δ*_C_		*δ*_H_
1	40.2		1.87 (*dt*, *J* = 3.2, 12.8), 1.09 (*m*)		35.3		1.91 (*dt*, *J* = 3.2, 12.1), 1.73 (*m*)
2	29.7		2.51 (*m*), 2.02 (*m*)		28.1		2.80 (*m*), 2.05 (*m*)
3	78.7		3.41 (*dd*, *J* = 12.0, 4.4)		71.1		4.75 (*brs, W_1/2_ = 5.7*)
4	49.6				48.8		
5	57.1		1.05 (*m*)		49.7		2.08 (*m*)
6	21.0		2.22 (*m*), 2.06 (*m*)		20.9		2.38 (*m*), 2.07 (*m*)
7	34.6		1.51 (*m*), 1.37 (*m*)		34.7		1.61 (*m*), 1.40 (*m*)
8	41.5				41.8		
9	50.8		1.29 (*m*)		50.8		1.53 (*m*)
10	37.5				38.7		
11	22.4		1.59 (*m*), 1.29 (*m*)		22.3		1.69 (*m*), 1.32 (*m*)
12	24.1		1.59 (*m*), 1.45 (*m*)		24.1		1.58 (*m*), 1.43 (*m*)
13	46.0		1.58 (*m*)		46.0		1.59 (*m*)
14	41.0				41.1		
15	32.8		2.18 (*m*), 1.53 (*m*)		32.7		2.18 (*m*), 1.51 (*m*)
16	120.9		5.34 (*d, J = 5.1*)		121.0		5.33 (*d, J = 5.0*)
17	139.7				139.7		
18	37.5				37.4		
19	38.9		1.61 (*m*), 1.18 (*m*)		38.9		1.60 (*m*), 1.18 (*m*)
20	35.8		1.53 (*m*), 1.18 (*m*)		35.8		1.51 (*m*), 1.16 (*m*)
21	33.0				33.0		
22	46.8		2.28 (*m*), 1.65 (*m*)		46.8		2.27 (*m*), 1.63 (*m*)
23	25.0		1.74 (*m*)		25.7		1.80 (*m*)
24	180.1				181.0		
25	15.1		1.11 (*s*)		14.9		1.21 (*s*)
26	17.3		1.03 (*s*)		17.5		1.09 (*s*)
27	16.7		1.06 (*s*)		16.7		0.97 (*s*)
28	18.0		1.00 (*s*)		18.0		0.97 (*s*)
29	25.0		0.84 (*s*)		25.1		0.83 (*s*)
30	32.8		0.96 (*s*)		32.8		0.95 (*s*)

Compounds **1**–**4** were evaluated fort their α-glucosiade inhibitory activity using *p*-nitrophenyl-*α*-d-glucopyranoside (PNPG) as the substrate [8]. Although, not stronger than the activity of the reference drug acarbose, these compounds still exerted mild inhibitory activity against *α*-glucosidase (Table 2).

**Table 2:** Inhibitory effects of compounds **1**–**4** and acarbose against *α*-glucosidase ^a,b^.

Compound	IC_50_ (μM)	Compound	IC_50_ (μM)
**1**	1932 ± 97	**4**	2017 ± 101
**2**	1812 ± 85	acarbose	627 ± 28
**3**	1858 ± 76		

^a^ IC_50_, the concentration that inhibits cell growth by 50%; ^b^ Each value represents the mean ± S.D. (n = 3).

## 3. Experimental

### 3.1. General Procedures

Optical rotations were measured on an AA10R digital polarimeter. IR Spectra were detected on Avater-360 spectrophotometer with KBr pellets, and are reported in cm^−1^. 1D and 2D NMR spectra (all in C_5_D_5_N) were recorded on a Bruker AV-400 spectrometer, and chemical shifts are expressed in δ (ppm) and referenced to the solvent peaks at δ_H_ (8.74, 7.59, 7.22) and δ_C_ (150.3, 135.9, 123.9) for C_5_D_5_N, respectively, and coupling constants are in Hz. HR-ESI-MS were determined on a Agilent 6520 Q-TOF LC-MS mass spectrometer. Semi-Preparative HPLC was performed on a Hitachi Spectra Series HPLC system equipped with an L-2130 pump and a UV L-2400 detector in a YMC-ODS column (10 mm × 250 mm, 5 *μ*m; flow rate at 2.0 mL/min; wavelength detection at 208 nm; retention time 34.2 min for **1**, 38.0 min for **2**, 18.7 min for **3**, and 20.1 min for **4**). Column chromatography (CC) was performed on SiO_2_ (200–300 mesh, Qingdao Marine Chemical Factory, Qingdao, China) and Toyopearl HW-40C (Tosoh Bioscience Shanghai Co., Ltd., Shanghai, China). Analytical TLCs were run on silica gel plates (GF_254_, Yantai Institute of Chemical Technology, Yantai, China). Fractions were monitored by TLC, and spots were visualized by heating TLC sprayed with 10% H_2_SO_4_.

### 3.2. Plant Material

The whole plant of *S. kouitchensis* was collected in Enshi, Hubei province, China, in October 2010, and identified by Prof. Jiachun Chen (Tongji Pharmaceutical School of HUST, Wuhan, China). A voucher specimen (*S.k*-2010-1010) has been deposited in the University herbarium for future reference.

### 3.3. Extraction and Isolation

The chopped, dried whole plants of *S. kouitchensis* (15 kg) were refluxed twice with 120 L of 95% (v/v) EtOH–H_2_O, two hours each time. After filtration, the filtrate was concentrated under reduced pressure to yield a brownish residue (3.0 kg). Part of the residue (2.5 kg) were suspended in water and partitioned successively with petroleum ether, CH_2_Cl_2_, EtOAc, and *n*-butanol to afford five fractions. The CH_2_Cl_2_-soluble part (about 400 g) was subjected to CC (SiO_2_, 200–300 mesh, 3.0 kg, 12 × 100 cm, petroleum ether/acetone 100:0→0:100) to yield five fractions A–E. Fraction B (87.4 g) was subjected to HW 40C (CHCl_3_/MeOH 1:1) to give four subfractions B_1–4_. B_2_ was subjected to CC (SiO_2_, CHCl_3_/ EtoAc 20:1→10:1) to give two subfractions B_2a_ and B_2b_. B_2a_ was purified by semi-preparative HPLC (MeOH/H_2_O 90:10) to yield compound **3** (78.5 mg) and compound **4** (13.0 mg). B_2b_ was purified by semi-preparative HPLC (MeOH/H_2_O 95:5) to yield compound **1** (4.1 mg) and **2** (5.7 mg).

*3β-Hydroxy-chirat-16-en-24-oic acid* (**1**). White amorphous powder. 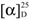 +67.2° (*c* = 0.3, pyridine); IR (KBr) *ν*_max_ 3453, 2940, 1696, 1461, 1379, 1035 cm^−1^; ^1^H-NMR and ^13^C-NMR see Table 1; HRESIMS *m/z* 455.3525 [M−H]^−^ (calcd. for C_30_H_47_O_3_, 455.3531).

*3α-Hydroxy-chirat-16-en-24-oic acid* (**2**). White amorphous powder; 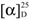 +35.3° (*c* = 0.2, pyridine); IR (KBr) *ν*_max_ 3448, 2934, 1701, 1456, 1378, 1036 cm^−1^; ^1^H-NMR and ^13^C-NMR see Table 1; HRESIMS *m/z* 455.3525 [M−H]^−^ (calcd. for C_30_H_47_O_3_, 455.3531).

### 3.4. *In Vitro* Inhibitory Activity against α-Glucosidase

*α*-Glucosidase (from *Saccharomyces cerevisiae*, Sigma-Aldrich, St. Louis, MO, USA) inhibitory activities were determined by using *p*-nitrophenyl-*α*-d-glucopyranoside (PNPG) as the substrate, according to the reported method [8]. Briefly, 20 μL of enzyme solution [0.6 U/mL α-glucosidase in 0.1 M potassium phosphate buffer (pH 6.8)] and 120 μL of the test compound in water containing 0.5% DMSO were mixed, and was preincubated for 15 min at 37 °C prior to initiation of the reaction by adding the substrate. After preincubation, PNPG solution 20 μL [5.0 mM PNPG in 0.1 M potassium phosphate buffer (pH 6.8)] was added and then incubated together at 37 °C for incubation. After the incubation, 80 μL 0.2 M Na_2_CO_3_ in 0.1 M potassium phosphate buffer was added to the test tube to stop the reaction. The amount of PNP released was quantified using a UVmax Kinetic Microplate Reader (Bio Tek, Synergy 2, Winooski, VT, USA) at 405 nm. 

## 4. Conclusions

Phytochemical investigation of CH_2_Cl_2_-soluble part of *S. kouitchensis* afforded two new chiratane-type triterpenoids, kouitchenoids A (**1**) and B (**2**), together with two known triterpenoids, oleanolic acid (**3**) and ursolic acid (**4**). Their structures were elucidated on the basis of spectral analysis and literature comparisons. All isolated compounds **1**–**4** exhibited moderate inhibitory activities against *α*-glucosidase *in vitro*, comparable with that of acarbose.
